# A Complex Network Approach for the Estimation of the Energy Demand of Electric Mobility

**DOI:** 10.1038/s41598-017-17838-5

**Published:** 2018-01-10

**Authors:** Mario Mureddu, Angelo Facchini, Antonio Scala, Guido Caldarelli, Alfonso Damiano

**Affiliations:** 1 University of Cagliari, Cagliari, Italy; 2 IMT School for Advanced Studies Lucca, Piazza San Francesco 19, 55100 Lucca, Italy; 3 CRN Institute for Complex Systems, via dei Taurini 19, 00185 Rome, Italy; 4 London Institute for Mathematical Sciences, 35a South Street Mayfair, W1K 2XF London, UK; 5 Linkalab, Complex Systems Computational Laboratory, Viale Elmas, 142 09122 Cagliari, Italy

## Abstract

We study how renewable energy impacts regional infrastructures considering the full deployment of electric mobility at that scale. We use the Sardinia Island in Italy as a paradigmatic case study of a semi-closed system both by energy and mobility point of view. Human mobility patterns are estimated by means of census data listing the mobility dynamics of about 700,000 vehicles, the energy demand is estimated by modeling the charging behavior of electric vehicle owners. Here we show that current renewable energy production of Sardinia is able to sustain the commuter mobility even in the theoretical case of a full switch from internal combustion vehicles to electric ones. Centrality measures from network theory on the reconstructed network of commuter trips allows to identify the most important areas (hubs) involved in regional mobility. The analysis of the expected energy flows reveals long-range effects on infrastructures outside metropolitan areas and points out that the most relevant unbalances are caused by spatial segregation between production and consumption areas. Finally, results suggest the adoption of planning actions supporting the installation of renewable energy plants in areas mostly involved by the commuting mobility, avoiding spatial segregation between consumption and generation areas.

## Introduction

The expected electrification of mobility is viewed as a great step in the progressive increase of its sustainability. According to the International Energy Agency, achieving the goal of maximum 2 degrees increase in global temperature established by the Paris Agreement would mean that approximately 19% of the GHG reduction should be attained through the mitigation of transport emissions. In addition, as urbanization will increase in the next years, electric mobility especially in large urban areas like megacities will play a fundamental role^1^. In fact, in 2011 the world’s gasoline share for mobility in 27 megacities was about 9%, and this share is expected to increase further^2,3^. Consequently, an increase of GHG emissions and air pollution, as well as a growing dependency on fuel import is expected.

Use of alternative, environmentally friendly fuels and power trains are therefore a key strategy for heading towards sustainable transport systems, and, together with Electric Vehicles (EV), renewable energy sources are considered a further winning strategy to reduce the GHG emissions from cities. As the number of EV is expected to increase, given the potential high number of vehicles involved, a reliable planning and control of the charging infrastructure will be fundamental for its successful integration in the power system. In particular, a correct sizing of the needed resources, in term of electricity, charging stations, communication and management infrastructures must be based on the local mobility needs and on both the distribution infrastructures and electricity market constraints^4^. In fact, electricity infrastructures, for decades based on a hierarchical, fossil based model of production and distribution needs to be replaced out by a new, flexible model of generation and distribution whose solid grounds are based on renewable energy sources, integration, and decentralization. In addition, thanks to an increasing level of digitalization of infrastructures, the integration between distributed generation and electric mobility is of great benefit. If well planned, it can simultaneously contribute to emission reduction and to grid resilience due to the ability of electric vehicles to act as distributed storage for stabilizing the power fluctuations on distributed electricity systems^5–7^.

With regards to electric mobility initiatives, the existing literature^8–10^ primarily focuses on the evaluation of the impact of electrical mobility infrastructure on a metropolitan scale. This does not take into account the long range impacts on both the mobility infrastructure and the grid. In fact, high penetration of EV in densely populated areas could impact on the surrounding electricity transmission system, leading to systemic effects which are difficult to identify by limiting the analysis to the sole city boundaries.

In the present paper a multi-layered approach based on complex networks theory^11–13^ is presented. We study the co-existence of two different network infrastructural layers, energy and mobility, to estimate the impact of electric mobility on a regional scale, with the aim to quantify both short range and long range effects. The proposed methodology allowed to perform a scale analysis which takes into account a multi-layered infrastructure scheme of the problem. The proposed approach exploits census data regarding commuting to estimate the origin-destination matrix for the EV trips. Commuting is particularly important for EV mobility, because it it composed by short and medium range trips, which combines well with the limited ranges of EVs. Moreover, the proposed methodology relies on the map of solar and wind generation plants to identify the municipalities that are able to produce the low carbon electricity to support the mobility of EV on regional scale. Using these two different datasets, it is possible to estimate the energy need of charging services both on cities and on freeways, as well as the investigating of the possible integration with the local grid and renewable generation plants.

The proposed planning of electric mobility is performed on different scales, according to the availability of data (including census, the mobility network and the installed generation plants). It is arranged in three steps:

1. Data collection and geo-referencing by means of Geographic Information Systems (GIS).
2. Definition of the complex system able to describe the expected interaction between mobility and power grids. The proposed approach also includes the estimation of the expected traffic flows starting from census commuting data, integrated by the energy description of EVs.
3. Identification of the interactions between the layers of the system to quantify the impact of the energy needs of mobility on the power grid of the territory, in terms of the associated energy flows.

The methodology has been applied on one of the major islands of Italy, the region of Sardinia, considering a scenario of full deployment of electric vehicles to cover the need of commuting mobility on regional scale. Results show that the current RES production in Sardinia is able to cover the full deployment of electric mobility for commuting. Moreover, the network analysis of the regional commuting patterns shows that a limited number of municipalities behave as hubs of the regional traffic. Also, in the majority of cases the energy needs are negative in such areas, i.e. they need further power to sustain the recharge infrastructure. Finally, the optimization analysis of energy flows reveals both long range effects and spatial segregation phenomena affecting in the energy transmission system.

## Results

**Figure 1:**
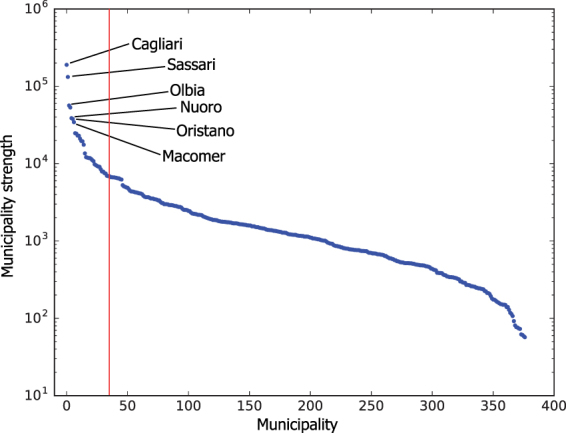
The plot shows the cumulative number of incoming and outcoming vehicles per each municipality. The red line indicated the more central municipalites, listed in Table 1.

**Figure 2:**
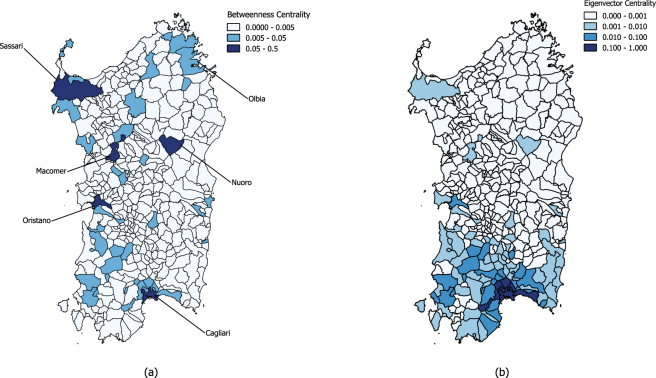
Georeferenced distribution of both the betweenness and eigenvector centrality of the municipalities of the commuting network of the island of Sardinia. Panel (a) refers to the betweenness centrality, while panel (b) refers to the eigenvector centrality. The maps have been produced with QGIS version 2.18.4^15^.

The first step of the proposed method is to identify the municipalities more involved in the commuting mobility: the origin-destination matrix is estimated by building a directed weighted network starting from the census data (see further details in the methods section). In the proposed representation the municipalities are the nodes of the network, links model the trips and weights correspond to the number of vehicles crossing the municipality borders (both incoming and outcoming flows is considered). From Table 1 and Fig. 1 it is possible to notice that a limited amount of municipalities concentrate a large share of trips, suggesting to use centrality measures to identify the most important areas. Figure 2 shows the results of the analysis computed with two methods (darker areas correspond to higher values): panel (a) shows the betweenness centrality, while panel (b) the eigenvector centrality. These measures offer two slightly different readings: the eigenvector centrality identifies the southern municipalities as the most central ones, while the municipalities highlighted by the betweenness centrality are distributed on the region, reflecting the distribution of the principal urban areas in Sardinia. Such difference is mainly due to the large concentration of high strength municipalities (i.e. those showing the higher number of connections and high strength nodes) located in the southern part of Sardinia, where the main city Cagliari is also located. Such distribution of high degree nodes has a relevant impact on the eigenvector centrality, and is also reflected in the high energy flows, as we will show later in this section. On the other side, the same effect does not stand for betweenness centrality, that also put in better evidence the municipalities located in the central and northern areas, leading to a more accurate identification of the central nodes. However, both measures are useful and highlight the role of the main municipalities located in Sardinia: Cagliari, Nuoro, Oristano and Sassari. On the other hand, the role of Olbia, another important city, is put in evidence only with the betweenness centrality measures. It is also worth noting that the reconstructed origin destination matrix confirms Macomer (a smaller town with respect to the other mentioned) to play an important role as a hub for the transport network in Sardinia (see Fig. 1). From the census data it is shown that the above mentioned municipalities concentrate the flow of about 455000 vehicles, as showed in Table 1 reporting the number of incoming and out-coming vehicles for the first 35 municipalities, chosen by setting a threshold *B* > 0.005 on the nodes centrality. Furthermore, this subset (9% of the total municipalities) concentrate the 64% of the trips, corresponding to a energy consumption of the 83% of the total (730 MWh). Moreover, the eigenvector centrality highlights the central role of south Sardinia in commuting, due to the high number of connections with the capital city, Cagliari.

**Table 1:** Number of daily incoming and outgoing vehicles in the 35 major municipalities of Sardinia.

Municipality	Incoming	Outgoing	Total	Energy Balance (kWh)
CAGLIARI	60968	33745	94713	320541
SASSARI	36793	29220	66013	34374
OLBIA	15110	13104	28214	36252
QUARTU SANT’ELENA	10296	16313	26609	−17734
NUORO	10977	8374	19351	4722
ORISTANO	11865	7059	18924	31023
ALGHERO	8519	8672	17191	−4948
SELARGIUS	4897	7466	12363	5118
IGLESIAS	6154	6057	12211	30747
ASSEMINI	4795	6728	11523	32934
CARBONIA	5786	5699	11485	1255
SESTU	5069	5454	10523	30201
PORTO TORRES	4902	4916	9818	167157
MONSERRATO	4914	4798	9712	−5828
CAPOTERRA	2980	5794	8774	5840
TEMPIO PAUSANIA	3644	3133	6777	6949
ELMAS	3736	2316	6052	3876
VILLACIDRO	3027	2953	5980	82152
ARZACHENA	3297	2613	5910	5043
SINNAI	1951	3951	5902	−491
TORTOLI’	3391	2385	5776	16775
MACOMER	3329	2229	5558	44170
OZIERI	2881	2504	5385	83428
SINISCOLA	2529	2370	4899	16663
QUARTUCCIU	1579	3198	4777	−2572
GUSPINI	2250	2421	4671	178701
LA MADDALENA	2266	2312	4578	504
SORSO	1554	2996	4550	−1404
SANLURI	2357	1872	4229	12867
SANT’ANTIOCO	1917	2035	3952	589
TERRALBA	1781	2163	3944	22996
DECIMOMANNU	1898	1876	3774	4017
SAN GAVINO MONREALE	2012	1708	3720	−214
DORGALI	1638	1847	3485	2949
DOLIANOVA	1229	2221	3450	183

**Figure 3:**
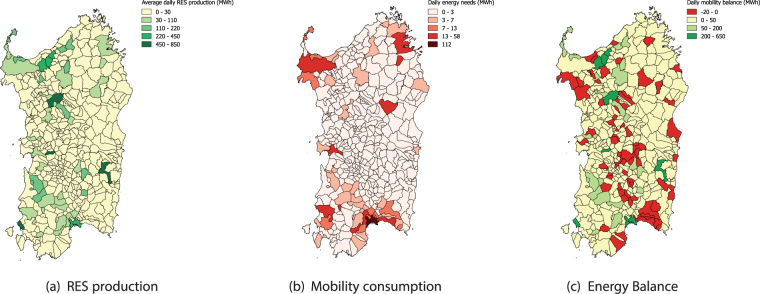
Georefenced distribution of average daily power production and estimated EVs electricity demand per each municipality of the case study. Distributed generation by RES is shown in green scale, whereas consumption is shown in red scale. The energy balance between distributed generation and electricity EV demand for each municipality is shown for the municipalities listed in Table 1. Municipalities in red colors show negative negative energy balance (i.e. net consumption), while green municipalities show a positive balance (i.e. a surplus in net production). The maps have been produced with QGIS version 2.18.4^15^.

Once identified the key municipalities involved in the commuting flows, a second layer is considered, introducing the energy balance between the distributed electricity production and the estimated consumption associated to the charging processes of EVs. The maps are showed in Fig. 3. Particularly, panel (a) shows for each municipality the Renewable Energy Sources (RESs) production, being the main form of distributed generation in all Sardinian municipalities, while panel (b) shows the electricity demand associated to EV commuting mobility.

The energy needs are computed considering three different charging behaviors (further details in the methods section) and refer to a full EV deployment scenario, accounting for about 700.000 vehicles belonging to the type specified in Table 2. By comparing Fig. 3a and b it is possible to notice how in some cases the electricity demand matches the corresponding area, while in the majority of cases the energy needs are not matched and lead to an energy imbalance. In particular, the electricity balances between RES production and EV demands of the larger municipalities are numerically reported in Table 1. It is worth noting by the analysis of the maps reported in Figs 2 and 3 that the most central municipalities (dark blue ones in 2a), have enough average local and distributed daily energy generation to support (from yellow to green in Fig. 3c) their EV fleet. On the other hand the same Fig. 3c highlights how the relevant unbalances are generated in the municipalities surrounding the main cities, revealing that the most unbalanced regions are found around Cagliari, the capital city of Sardinia, and Sassari.

**Table 2:** Energetic characteristics of the vehicles considered in the scenario and composition of the fleet^16^.

Vehicle	Capacity (kWh)	Autonomy (km)	Estimated consumption $(\tfrac{{\boldsymbol{kWh}}}{{\boldsymbol{km}}})$	Fleet composition
Nissan Leaf	24	199	0.12	80%
Volkswagen E-Golf	24	190	0.127	15%
Tesla Model S	85	491	0.173	5%

**Figure 4:**
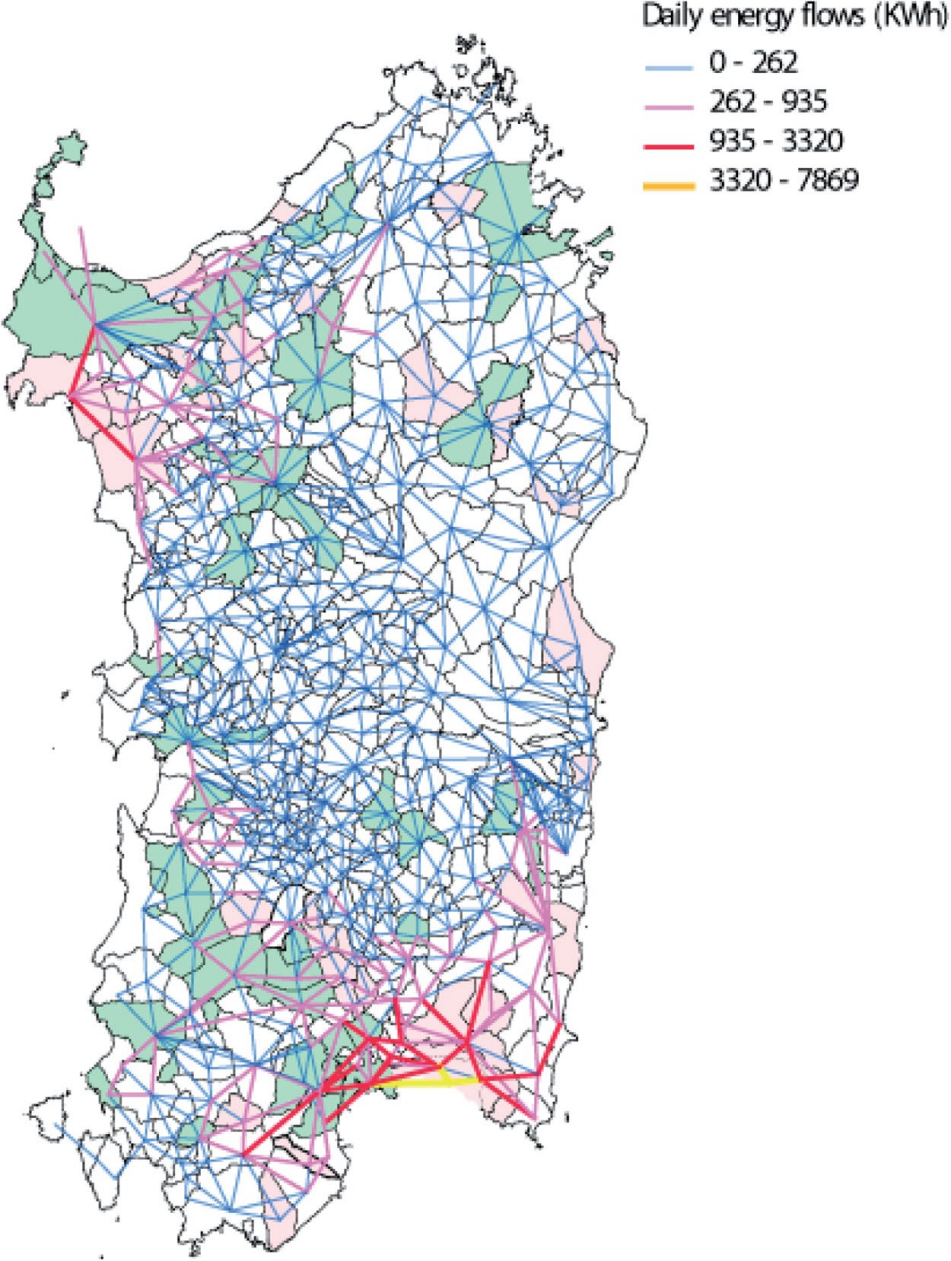
Geographical representation of the daily energy flows between each municipality. Relevant energy flows are present in the southern part of the Island, where the municipality of Cagliari is located. Map colors indicate the municipalities listed in Table 1 showing negative or positive (i.e. surplus) in the energy balance. Red means negative balance, while green is positive. The high flows (red) are mainly originated by the spatial segregation between production and consumption areas. Long range effects are visible in the areas surrounding Cagliari and Sassari (north-west), and are colored in pink (262–935 kWh). The map has been produced with QGIS version 2.18.4^15^.

As a further step, the impact of the mobility infrastructure on the power grid is quantified, by computing the daily energy flows associated to the energy unbalances. This has been achieved by solving the flow equation presented in methods section, with the aim to ensure local power balance in each node minimizing the transmission costs over the power network. Figure 4 shows the energy flows caused by the unbalances. The flows range from 0 to 7800 kWh and green municipalities indicate the ones contributing more to the energy production, while red municipalities are those showing unbalances up to 20 MWh (see Fig. 3c). As a result, it is possible to notice how the mobility needs of the two biggest cities of the territory can cause high energy flows in the regional grid. Especially the southern zone, highly connected with the capital city of Cagliari, shows significant energy transactions which extend up to 100 km from the traffic attractor. This effect is also suggested by the eigenvector centrality analysis in the Fig. 2b.

## Discussion

The proposed method aims to assess the impact on regional scale of electric mobility fueled by low carbon sources. It is organized in the following steps:

1. Reconstruction of the origin-destination matrix by means of census data.
2. Use of complex networks to identify the most important areas interested by the recharge infrastructures.
3. Modeling the behavior of commuters.
4. Optimization of flows in order to minimize the energy transmission costs

According to the presented results, the proposed method suggests that an accurate spatial balance between production and consumption areas is needed in order to avoid relevant energy flows that negatively impact on the transmission network. Furthermore, the results can be used to identify the position of charging stations and RES production areas at regional level.

The analysis of the flows clearly shows how the highly populated areas (here, Cagliari and Sassari, see Fig. 2a) impact the regional power grid even in regions far from their respective areas, suggesting systemic effects on the multilayer system. Since these long range effects can be detrimental for the power grid operation, this clearly shows how a multilayer analysis on a large scale is necessary to fully understand the impact of electrical mobilities in big cities.

According to the general balance between distributed generation and electric mobility consumption, it is important noting that the actual production of the whole region is able to sustain the full deployment of EV for commuting with a limited stress on the distribution system.

The expected electrification of mobility is viewed as a great step in the progressive increase of its sustainability. Moreover, the use of parked Electric Vehicles (EVs) implementing the Vehicle to Grid technology as distributed storage systems is viewed as a way to limit the impact of the intermittent power output typical of Renewable Energy Sources (RES). This will lead to a gradual integration between mobility and smart grids, also leading to the implementation of Virtual Power Plants in areas where charging stations are concentrated (e.g., parking spaces in connection with public transport stops).

Future research will take into account the temporal patterns of the incoming and outcoming vehicles, considering their impact of the charging and discharging process on the main grid. Furthermore, the direct interaction of energy and mobility will be studied by using the theoretical results obtained in the field of multiplex networks, with the aim to consider the integration of the RES in the grid and to include in the energy flows the possibility to use vehicle to grid strategies to further reduce the impact on the network.

## Methods

The calculation of energy impact on a regional scale is based on the public available data regarding mobility and RES production, and relies on the knowledge of two key quantities: the mobility network, represented by its adjacency matrix **A**, and the distributed energy production ${P}_{m}^{\ast }$ of each municipality *m*. A detailed description of the procedures used for the determination of these quantities is given in the data subsection.

Starting from these two quantities, the daily consumption of charging of EV batteries is calculated, starting from the commuting network. The approach consists in associating an energy weight *W*_*ij*_ to the commuting links, as described in 1. Here, *d*_*ij*_ and *N*_*ij*_ are the driving distance and the number of trips between municipality *i* and *j*, respectively. $FC=0.123\,\tfrac{kWh}{Km}$ is the energy consumption of each vehicle, estimated from a fleet composed by 80% of Nissan Leaf, 15% of VW E-Golf, and 5% of Tesla Model S. Their characteristics are given by the constructor and shown in Table 2. The network allows self-loops, and in this case the distance *d*_*ii*_ is calculated as the size of the town/city.

1 $${W}_{ij}={d}_{ij}\cdot FC\cdot {N}_{ij}$$

The calculation of the energy consumption ${C}_{m}^{\ast }$ of each municipality *m* is performed by assuming the existence of a diffused charging infrastructure which allow the users to charge their vehicles both at home and at the workplace. Therefore, it has been assumed that the drivers can fall in one of three different categories: home charger, work charger and regular charger. Home chargers charge only at home, work chargers charge only at workplace, and regular chargers charge half at home and half at workplace. In the model, each category is represented in the population by the values *f*_*WC*_ *f*_*CC*_ and *f*_*HC*_, respectively. In this paper, ${f}_{WC}={f}_{CC}={f}_{HC}=\tfrac{1}{3}$.

In this way, the energy need *C*_*i*_ of each node *i* can be calculated as eq. 2.

2 $${C}_{i}=\sum _{j}\,({f}_{HC}+\tfrac{1}{2}{f}_{CC}){W}_{ij}+\sum _{j}\,({f}_{WC}+\tfrac{1}{2}{f}_{CC}){W}_{ji}+{W}_{ii}$$

The power balance *B*_*m*_ of each municipality *m* is then calculated as 3.

3 $${B}_{m}^{\ast }=\sum _{t}\,{P}_{mt}^{\ast }-{C}_{m}^{\ast }$$

The power flow optimization is based on the assumption that each municipality has a potential *V*_*i*_, that will define the incoming and outcoming flows with the neighbors. The values of *V*_*i*_ will be obtained by solving the system of equations shown in eq. 4, where *E*_*ij*_ = *K*_*ij*_ · (*V*_*i*_ − *V*_*j*_).

4 $${B}_{i}-\sum _{j}\,{E}_{ij}=\mathrm{0,}$$

The system of equations in 4 can be easily solved in matrix form, by

$${\bf{B}}-{\bf{K}}\cdot {\bf{V}}=0,$$

where **B** is the vector of balances *B*_*i*_, **K** is the matrix of couplings of the system, and **V** is the vector of *V*_*i*_. **K** is given by the entries *K*_*ij*_, representing the couplings between node *i* and *j* of the network. It has been obtained by the following eq. 5, where D is a diagonal *L* × *L* matrix representing the lines couplings. In this case, the matrix D is choosen as the identity matrix, representing equal coupling among the nodes. For this reason, the elements *V*_*i*_ are obtained from the equation 6.

5 $$K={A}^{T}DA$$

6 $${\bf{V}}={{\bf{K}}}^{-1}\cdot {\bf{B}}$$

Since, in general, the available resources are higher than the power needs, it is necessary to define a univoque way to allocate the resources. This is done by obtaining the solution that minimizes the quality function in eq. 7, where **B** is a vector containing all the balances *B*_*i*_ of the municipalities.

7 $$f({\bf{B}})=\sum _{i,j}\,{({V}_{i}-{V}_{j})}^{2}$$

The solution should be calculated under the constraint given in eq. 8, which represents the balance equation of the system, where ${B}_{i}={P}_{i}-{C}_{i}^{\ast }$, being *P*_*i*_ the power output of municipality *i*. Also, the values *B*_*i*_ should respect the inequalities $-\,{C}_{I}^{\ast }\le {B}_{i}\le {P}_{i}^{\ast }-{C}_{I}^{\ast }$. Given the defined quantities and constraints, the function *f*(**B**) has been minimized by means of the Sequential Least SQuares Programming (SLSQP) minimization procedure^14^. The solution of the minimization problem returns the vector **B**_*min*_. From this vector, it is easy to obtain the system flows *E*_*ij*_, and the power provided by each municipality ${P}_{i}={B}_{I}-{C}_{i}^{\ast }$.

8 $$\sum _{i}\,{B}_{i}=0.$$

### Data and case study

The proposed methodology has been applied on the Sardinian region, in Italy. The commuting dataset has been obtained from the Italian Census data, available at the ISTAT website http://www.istat.it/en/. The dataset contains the commuting origin/destination matrix of the territory, for an average workday. The dataset distinguish between trips performed by car, bus, train, bikes, bicycles, and other means of transportation. Also, in the car category, the dataset distinguishes between car drivers and car passengers, allowing to identify the number of vehicles moving from each municipality to another one. Also, the dataset contains the expected trips per different hours of the day, which have been here aggregated for the whole day. The dataset can be represented by means of a directed weighted graph described by its adjacency matrix *A*. The entries *a*_*ij*_ of the adjacency matrix are given by the number of trips occurring from municipality *i* to municipality *j*. The total number of trips provided by the dataset is 720200.

The power produced by RES in each municipality of the territory can be estimated from public datasets. The information on the wind and PV plants installed in Italy can be found on websites Atlasole (http://atlasole.gse.it/atlasole/) and Atlaeolico (http://atlanteeolico.rse-web.it/). The information provided for each plant includes the municipality in which it is installed, the nominal capacity and the year of installation. Also, information about the capacity factor is given. This information can be spatially aggregated, and the total installed power *P*_*m*,*t*_ can be calculated for each municipality *m* and RES technology *t*. This quantity is calculated by using eq. 9, where ${P}_{m,t}^{inst}$ is the aggregated installed capacity, and ${k}_{m,t}^{fact}$ are the capacity factors of wind and PV in each municipalities *m* and technology *t*.

9 $${P}_{m,t}={P}_{m,t}^{inst}\cdot {k}_{m,t}^{fact}\cdot 24.$$
